# Global perspectives on *CYP2D6* associations with primaquine metabolism and *Plasmodium vivax* radical cure

**DOI:** 10.3389/fphar.2022.752314

**Published:** 2022-11-15

**Authors:** Jasmine M. Olvany, Scott M. Williams, Peter A. Zimmerman

**Affiliations:** ^1^ The Center for Global Health and Diseases, Pathology Department, Case Western Reserve University, Cleveland, OH, United States; ^2^ Department of Genetics and Genome Sciences, Case Western Reserve University School of Medicine, Cleveland, OH, United States; ^3^ Department of Population and Quantitative Health Sciences, Case Western Reserve University School of Medicine, Cleveland, OH, United States; ^4^ Master of Public Health Program, Case Western Reserve University, Cleveland, OH, United States

**Keywords:** malaria, hypnozoite, CYP2D6, activity score, primaquine

## Abstract

Clinical trial and individual patient treatment outcomes have produced accumulating evidence that effective primaquine (PQ) treatment of *Plasmodium vivax* and *P. ovale* liver stage hypnozoites is associated with genetic variation in the human cytochrome P450 gene, *CYP2D6*. Successful PQ treatment of individual and population-wide infections by the *Plasmodium* species that generate these dormant liver stage forms is likely to be necessary to reach elimination of malaria caused by these parasites globally. Optimizing safe and effective PQ treatment will require coordination of efforts between the malaria and pharmacogenomics research communities.

## Introduction—malaria, primaquine and human genetics

In 2021, 241 million clinical cases and approximately 627,000 deaths were attributed to malaria (World Health Organization, 2020). *Plasmodium vivax* and *P. falciparum* are the most prevalent human malaria parasites, with an estimated 2.5 billion people at risk (Gething et al., 2011; Gething et al., 2012). While *P. falciparum* is currently recognized as the most lethal malaria parasite, *P. vivax* is the most geographically dispersed species. Furthermore, a now well-established body of literature describes severe, life-threatening clinical illness caused by *P. vivax* involving severe anemia (Tjitra et al., 2008), respiratory distress (Genton et al., 2008; Fernandez-Becerra et al., 2009), liver dysfunction (Barcus et al., 2007; Kochar et al., 2009), and renal failure (Siqueira et al., 2010). *P. vivax*-attributable mortality has been reported and confirmed globally (Beg et al., 2002; Kochar et al., 2005; Genton et al., 2008; Tjitra et al., 2008; Alexandre et al., 2010). In addition to the contribution that *P. vivax* contributes to significant morbidity and mortality, infections come with special complexity because the parasite can form a dormant hypnozoite stage in the liver that does not cause illness, but can re-emerge to cause a relapse infection from weeks to more than 1 year later, without mosquito transmission (Krotoski et al., 1982; Krotoski, 1985, 1989). Therefore, eliminating the hypnozoite(s) from infected people, termed radical cure, is essential for controlling *P. vivax* malaria. Achieving this requires a specific treatment as almost all approved antimalarials lack activity against hypnozoites (Thriemer et al., 2017). If *P. vivax* infection is treated without additional targeting of the liver-stage hypnozoite, the parasite is free to cross a broad geographic range, to persist across seasonal changes where mosquito vectors are absent, and to cause repeated clinical attacks, resulting in substantial cumulative morbidity. Thus, the chronic nature of *P. vivax* infections, mediated by hypnozoites, means that this parasite’s burden is harder to estimate (Anstey et al., 2012; Battle et al., 2016). Radical cure of *P. vivax* at the population level, therefore requires eliminating the hypnozoite reservoir to reach worldwide malaria elimination.

Currently, two drugs can kill hypnozoites and achieve radical cure, primaquine (PQ) and tafenoquine (TQ). Both drugs are 8-aminoquinoline (8AQ) derivatives (Wells et al., 2010; White, 2019). Although TQ has been approved by two national regulatory agencies (Australia in 2018; United States in 2019), PQ remains the only WHO-recommended drug to achieve radical cure (WHO approved in 1952 (Hill et al., 2006; World Health Organization, 2015). While effective against the liver stage hypnozoite, these 8AQ drugs are known to induce acute hemolytic anemia (AHA) in individuals who inherit the X-linked glucose-6-phosphate dehydrogenase enzyme deficiency (G6PDd); G6PDd is the most common human enzyme deficiency (Howes et al., 2012; Luzzatto et al., 2016), and it is in particularly high frequency in malaria endemic regions as it confers protection from severe malaria (Bienzle et al., 1979; Ruwende et al., 1995; Sirugo et al., 2014). As PQ is taken daily over 2 weeks (5 h half-life), treatment (0.25–0.5 mg base/kg body weight) can be stopped if any signs of AHA are observed; PQ can also be administered once each week over 8-week (0.75 mg base/kg body weigh) for G6PDd individuals (males or females with less than 30% G6PD enzyme activity (World Health Organization, 2016). Because of a much longer half-life (15 days), TQ is administered *via* a single dose, observation of AHA cannot be mitigated, and thus the elevated potential for harm to G6PDd individuals supports restricted use protocols and limited approvals (White, 2019). Notably, across the globe, G6PDd presents a barrier to universal implementation of mass drug administration against vivax malaria.

In the past 10 years, complexities of PQ pharmacogenetics have emerged as the therapeutic efficacy of PQ appears to be strongly correlated with activity of the highly polymorphic metabolic enzyme, cytochrome P450 2D6 (*CYP2D6*) (Bennett et al., 2013; Ingram et al., 2014; Silvino et al., 2016; Baird et al., 2018b; Brasil et al., 2018), an enzyme implicated in metabolism of >20% of marketed drugs (Gaedigk et al., 2018a). PQ metabolism involves three predominant pathways: 1) glucuronide/glucose/carbamate/acetate conjugation; 2) hydroxylation at multiple positions on the quinoline ring; and 3) oxidative deamination at the terminal amine of the aminoalkyl side chain (Avula et al., 2018). Despite increasing knowledge of PQ metabolism and how it is affected by CYP2D6, the active anti-malarial compound has still not been defined with certainty, although data now indicates activity is mediated through hydroxylated metabolites whose formation is CYP2D6 dependent (Camarda et al., 2019). In contrast, while pharmacogenetic studies focused on TQ have suggested no association with CYP2D6, only limited insight into the metabolism of this drug exists based on a single study (St Jean et al., 2016).

Building a global population genetic picture of *CYP2D6* variation and how it relates to safety and effectiveness of 8AQ treatment is critical due to the potentially large hypnozoite reservoir in malaria, but also because these drugs can also play a role in limiting *P. falciparum* transmission through gametocytocidal activity (Single low dose PQ (0.25 mg/kg) blocks *P. falciparum* gametocyte transmission without adverse reactions in G6PDd individuals (Bancone et al., 2016)). Here we focus on current knowledge of population genetic variation of *CYP2D6* and PQ metabolism in hopes of expanding the population that can use this drug effectively (Baird et al., 2018a) to reduce the hypnozoite reservoir. This is consistent with recent clinical trials to test optimal dosing strategies against relapse infections (Brito-Sousa et al., 2022; Chamma-Siqueira et al., 2022). An adjoining article by Stewart et al. focuses on implications of *CYP2D6* and *G6PD* genetic variation on potential treatment approaches and limitations for *P. vivax* as well as *P. ovale* (that also produces hypnozoites) liver stage parasites (Stewart et al., 2021).

## CYP2D6 genetic variation and metabolic Activity Scores

*CYP2D6* is the most polymorphic of the *CYP* genes (Zhou et al., 2017). Currently, there are 145 known major “star” (*) alleles and multiple sub-alleles that include at least 128 unique SNPs (upstream non-coding and coding region), 7 insertions (one to multiple nucleotides or duplicated nucleotides), 7 deletions (1 to multiple nucleotides), 5 gene deletions, gene duplication, conversion or hybridization variations that can together generate more than 1,220 unique genotypes (Gaedigk et al., 2018b; Luo et al., 2021) (ongoing updates can be followed at https://www.pharmvar.org/gene/CYP2D6). Many of the mutations are quite rare (e.g. occurring in single individuals or isolated populations (Luo et al., 2021) and a significant number of resulting * alleles occur at frequencies less than 0.25%, and hence do not substantially affect drug metabolism at the population-level. That said, we have shown, in a study of *CYP2D6* in a Malagasy population, that the unexpected appearance of some alleles associated with diverse populations, including new alleles, new allele frequency patterns, genotype combinations and genotype proportions may reveal population level patterns of special relevance to malaria treatment (Mehlotra et al., 2021; Chan et al., 2022).

**FIGURE 1:**
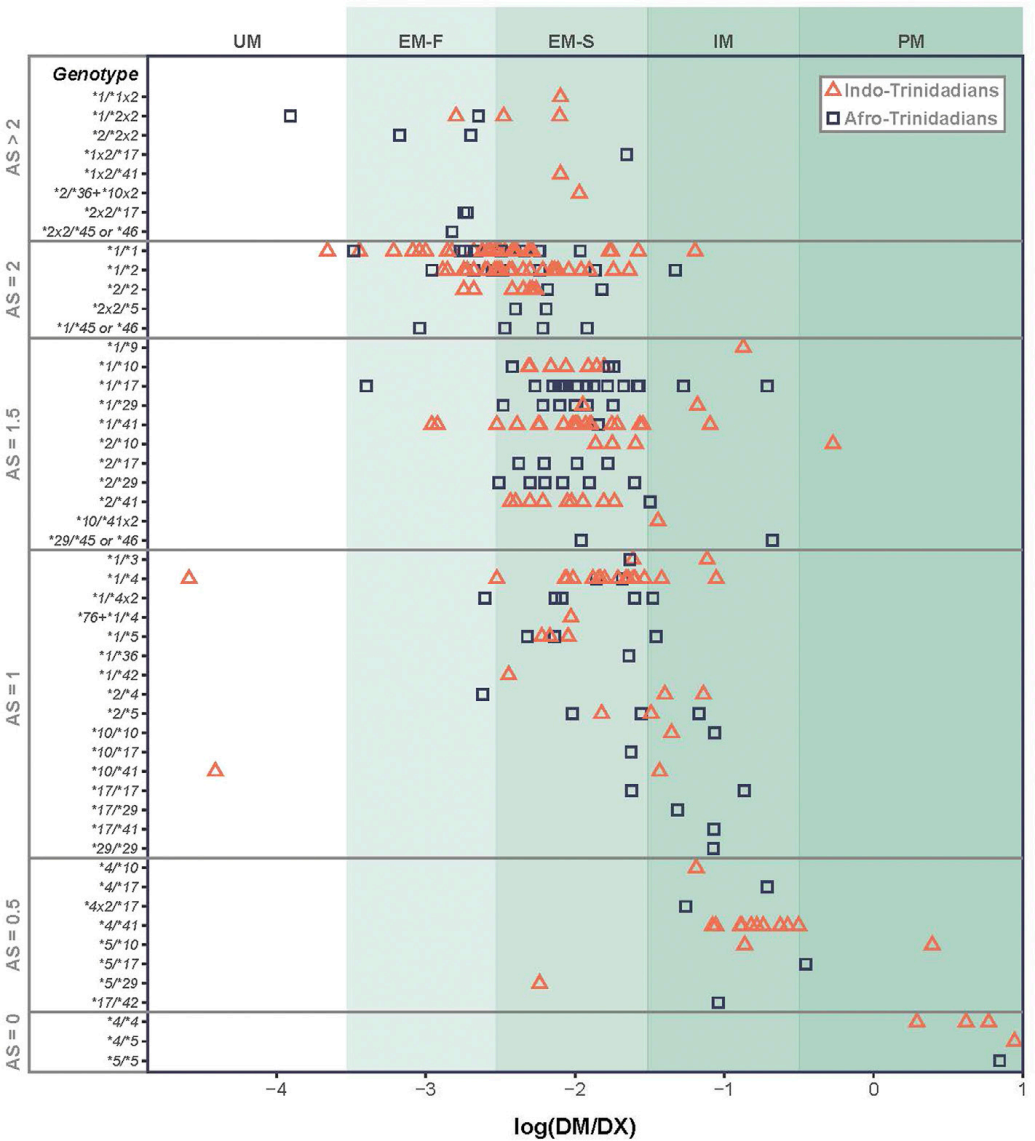
Variation in metabolism of dextro-methorphan (DM) to dextrorphan (DX) as a function of CYP2D6 * diplotypes. Diplotypes of the same AS and/or * designation can vary in metabolic rate by orders of magnitude, giving rise to ambiguity of our understanding of likelihood of radical cure based on these criteria.

Gaedigk et al. (2008) and PharmVar collaborators have further classified *CYP2D6* function through metabolism of dextro-methorphan (DM) to dextrorphan (DX). They correlated * allele genotypes with DM/DX ratio to define an Activity Score (AS) metric. Although the AS likely represents a good approximation of the generation of active anti-malarial compounds, the challenge of associating *CPY2D6* sequence variation with phenotypic effects is well appreciated (Gaedigk et al., 2018a; Pey, 2020). One example illustrating the complexity of the relationship between *CYP2D6* genotype and AS was observed in a study of 270 Trinidadians by Montané et al. and summarized in Figure 1 (Montane Jaime et al., 2013) Herein, the most regular association between genotype and AS was among individuals who were homozygous for the same, or heterozygous for different nonfunctional alleles, *4 and *5, as they clearly were poor metabolizers (i.e., AS = 0). For other allelic combinations the average DM metabolism is observed to increase with genotypes categorized with increasing AS (Montane Jaime et al., 2013). However, the variance of metabolism can be very large within functional * allelic categories, as individuals with the same *CYP2D6* genotype can differ in their metabolism of (DM) by up to 4 orders of magnitude (e.g. *1/*1; *1/*4) (Montane Jaime et al., 2013). Gaedigk et al. have discussed several factors that contribute to this AS variability, including SNPs influencing *CYP2D6* gene and protein expression. They further call attention to missing or incomplete genetic information—where low or no-function alleles can be incorrectly assigned as *1 or *2 alleles, if specific SNP positions are not included in the genotyping strategy used (Gaedigk et al., 2018a). Of additional relevance, the AS of low activity CYP2D6*10 has recently downgraded to 0.25 (Caudle et al., 2020; Crews et al., 2021). These sources of genetic variation within *CYP2D6* and genetic polymorphisms across the human genome create the potential to modify the phenotypic AS for probe drugs as well as drugs to treat malaria and other wide-ranging health conditions (implications are focused on PQ discussed in sections below).

## Pharmacogenomics and CYP2D6 phenotype

Pharmacogenomics studies are certain to improve as large-scale sequence data are associated with phenotypic variation. Developing optimal study designs will require considerable discussion and beginning this process has been a motivation for this perspective. Cohort characteristics (size, gender, age-groups, and ethnicities) and sequencing strategies are key elements to consider. Ensuring sufficient power for detecting statistically significant associations between *CYP2D6* genotype, genetic modifiers and AS are necessary to improve specific drug effectiveness on the population-level.

Recent efforts have begun to address the patterns of genetic variation in *CYP2D6,* although given the level of polymorphism in this gene they are so far limited in their scopes, sizes and generalizability (Del Tredici et al., 2018; Puaprasert et al., 2018; Nguyen et al., 2019; Chan et al., 2022). Cramer (Cramer, 2019) has provided information of how a comprehensive genomic approach can enhance our understanding of CYP2D6 pharmacogenomics. Key to future studies will be the need to determine if a limited number of SNPs can be converted into more tractable methods for assessing *CYP2D6* genetic predictions of AS and whether these metrics relate only to a specific population. It will be important to determine if the same * allele in different populations is associated with AS the same functional variation, and whether genotype-phenotype association differences are based on unknown SNPs within *CYP2D6* or result from unique distributions of modifiers in other genes distributed throughout the human genome.

With these topics in mind, 1,000 Genomes data, have been used in two studies to illustrate population characteristics of CYP2D6 variation in association with geographic distribution of P. vivax malaria (Puaprasert et al., 2018; Cramer, 2019). Puaprasert showed that simply increasing geographic distance within Asia from their sampled Karen population on the Thai-Myanmar border correlated with increasing genetic differentiation at this locus, indicating that the transferability of genetic information among diverse populations will likely be limited (Puaprasert et al., 2018). Cramer has used this global resource to illustrate first how null and low activity CYP2D6 alleles and genotypes are dispersed throughout the world and further characterize a Malagasy population studied by Mehlotra (Mehlotra et al., 2021) to illustrate how genetic admixture between Asian and African superpopulations may have affected *CYP2D6* * allele distributions in this understudied population (Mehlotra et al., 2021). They further used the pattern of *CYP2D6* dispersal to hypothesize how PQ may be metabolized and hence used to treat malaria in different populations. Resulting predictions of activity indicate that the use of PQ following current protocols may be more effective in some malaria-endemic regions than in others as a result of high frequencies of poor metabolizer alleles in East Asia. Stewart et al. have called further attention to similar population-specific concerns in Central America as summarized previously (Stewart et al., 2021).

A recent clinical study of 57 Javanese male soldiers who had *P. vivax* malaria and received the same PQ regimen has provided insight into the complexities of *CY2D6* genetic polymorphism, associated AS and *P. vivax* relapse. Among 21 individuals who relapsed, AS score was <1.5 for 18 and ≥1.5 for 3 individuals whereas of the 36 who did not relapse 14 had an AS <1.5 and 22 had an AS of ≥1.5. The results indicate that *CYP2D6* genotypes associated with but did not completely predict PQ efficacy against *P. vivax* relapse. The AS did have utility, but the study emphasized the need to assess how additional variation at *CYP2D6* and elsewhere in the genome, can contribute to PQ metabolism and treatment efficacy. That the role of genotypes on radical cure is less than straightforward was further shown in a study conducted by the Australian Defence Force who returned from Papua New Guinea and East Timor (Chen et al., 2019). In this study, AS was not associated with radical cure, except in the case of non-functional alleles that associated with no cure.

Takeaways from these studies indicate that we need to better understand the allelic variation within single * alleles that are used to delineate AS variation and how patterns of these unknown genetic variants affect gene function. As a number of different sequencing and PCR-based strategies have been used to generate the data in these and other studies, limitations will be encountered in comparing genotype and phenotype associations both within and between studies. We also need to know how genetic variations within *CYP2D6* are distributed among human populations, as well as variation in the genes that interact with *CYP2D6*, to optimize PQ treatment and radical cure. Finally, it will be important to ask if and to what extent variation in *CPY2D6* genotype alters risks associated with G6PDd. Specifically, would AS <1 require more than 8 weeks of PQ treatment?

## External confounding factors

Beyond the intricacies of gene-specific and genome-wide variation, there are examples that other drugs, foods or herbal treatments can interact with PQ and influence biological phenotypes. Examples include other antimalarial drugs (e.g. chloroquine) (Pukrittayakamee et al., 2014; Fasinu et al., 2016), or medicinal herbal plants commonly found in the tropics and sub-tropics (e.g. *Hyptis suaveolens* (bush mint) (Thomford et al., 2018). It was observed that chloroquine (still used to treat *vivax* malaria) administration resulted in increased PQ plasma concentrations (Pukrittayakamee et al., 2014), and inhibited formation of several PQ metabolites predicted to be active against hypnozoites (Avula et al., 2018). Similarly, *H. suaveolens* can inhibit *CYP2D6* in a reversible and time dependent manner; therefore administration of PQ for radical cure needs to be done in full knowledge of these and likely other interacting compounds (Thomford et al., 2018).

We must also be mindful of the potential that PQ efficacy may be influenced by the parasite’s genetic constitution. Traditional approaches for verifying the effectiveness of antimalarial drugs includes testing genetic signatures of pre- and post-treatment infections using highly polymorphic parasite genes (e.g. circumsporozoite protein or merozoite surface protein (Imwong et al., 2007)). However, approaches based on only one or two genes limit the ability to distinguish multiple strains, and a broader genomics strategy has been proposed for detecting evidence of relapse (Popovici et al., 2019). Regardless of the molecular marker strategy to decipher strains in complex infections, assessing recrudescence, reinfection or relapse will continue to pose a significant challenge to studies monitoring drug effectiveness (Ferreira et al., 2021). For CYP2D6 studies this means that it will be important to determine if a relapse has resulted from lower-than-expected metabolism of PQ by the infected person, parasite resistance to PQ, and/or reinfection.

## Parting thoughts

**FIGURE 2:**
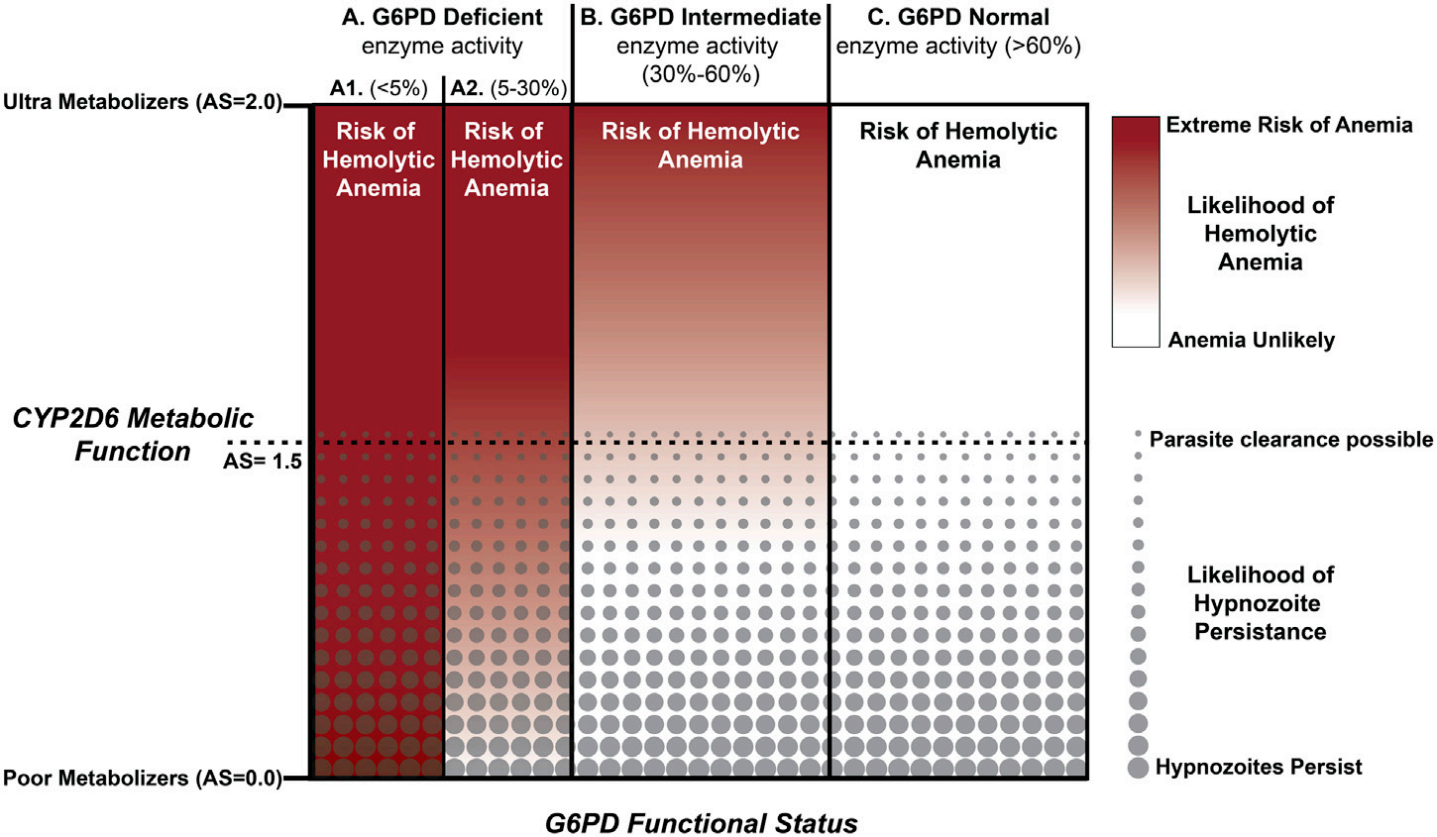
Integration of CPY2D6 Metabolic Function and G6PD Enzyme Activity. Those with a CYP2D6 AS < 1.5 are predicted to have an elevated risk of relapse. PQ failure with AS>1.5 appears to be less common and for those with AS > 2.0 radical cure success improves substantially following current standard of care. Integrated across the familiar G6PD enzyme activity benchmarks, potential exists for optimizing treatment strategies for all but those individuals with <5% G6PD enzyme activity.

The primary themes corresponding to genetic polymorphism associated with safety and effectiveness of PQ treatment are summarized in Figure 2 and frames the path forward for radical cure of the hypnozoite reservoir. Treatment safety is the main concern that has held back the use of PQ for radical cure of hypnozoite infection globally. Emerging point-of-care technologies that demonstrate capacity to perform quantitative assessment of G6PD enzyme activity, have shown promise in repeatability and reproducibility assessments (Ley et al., 2022). If greater precision can be achieved in determining G6PD enzyme activity at the point of PQ treatment, it may become possible to administer this drug safely to a many more G6PDd individuals (those with 5–30% enzyme activity; Figure 2, part A2). Better tools for determining G6PD activity would certainly increase confidence in treating those with 30–80% enzyme activity (Figure 2, part B). Regardless of G6PD enzyme activity, the remaining concern is whether sufficient concentrations of the pharmacologically active PQ metabolites will be generated to kill hypnozoites.

Finally, while current knowledge limits our ability to make inferences about appropriate dosing, at either the population or individual levels, it is important to continue to improve knowledge of the genotype to phenotype map so that we can more accurately predict PQ metabolism to provide more effective and safer treatment in line with current Clinical Pharmacogenetics Implementation Consortium (CPIC) guidelines (Crews et al., 2021). Simply, we need to consider how genetic and genomics technologies can drive the future of anti-malarial treatment as the motivation and ability to genotype is shifting rapidly. This is increasingly evident where malaria is endemic as reports now encourage expanding diversity in genomic studies (Sirugo et al., 2021; Krainc and Fuentes, 2022) and subsequent improved implementation (Wonkam, 2021; Chimusa et al., 2022). Many such practices are already in place in cancer studies around the world. As a result of these efforts, capacity to perform complex genotyping has advanced in fields of knowledge, attitudes and practice (KAP) (Muzoriana et al., 2017; Rahma et al., 2021; Koufaki et al., 2022), pharmacogenomics (Marsh et al., 2006), diabetes and hematology (Kountouris et al., 2021) globally. Additionally, studies focused on understanding patterns of genetic variation are advancing and will expand capacity to resolve their health implications in diverse populations (Taliun et al., 2021; DiCorpo et al., 2022). Given what we are observing across human genomics investigations, efforts are well underway to extend the breakthroughs available through genomics technologies as a global health mission and must be included as part of malaria elimination strategies.
